# Patellar resurfacing versus nonresurfacing in total knee arthroplasty: an updated meta-analysis of randomized controlled trials

**DOI:** 10.1186/s13018-020-02185-5

**Published:** 2021-01-25

**Authors:** Kai Chen, Xiaoyu Dai, Lidong Li, Zhigang Chen, Haidong Cui, Shujun Lv

**Affiliations:** 1 Department of Orthopedic Surgery, Hai’an People’s Hospital, Zhongba Road 17, Hai’an, Nantong, 226600 Jiangsu People’s Republic of China; 2 Department of Orthopedic Surgery, The First People’s Hospital of Changzhou Affiliated to Soochow University, Juqian Road 185, Changzhou, 213000 Jiangsu People’s Republic of China

**Keywords:** Patellar resurfacing, Total knee arthroplasty, Updated meta-analysis, Randomized controlled trial

## Abstract

**Background:**

Whether resurface the patella or not in total knee arthroplasty (TKA) was controversial. In 2013, we conducted a meta-analysis of randomized controlled trials (RTCs). After that, plenty of studies have been carried out, but there still existed a great deal of controversy. In order to update our previous study, we conducted this update meta-analysis to evaluate the efficacy of patellar resurfacing in TKA.

**Methods:**

Databases were searched for RCTs comparing the outcomes of patellar resurfacing and nonresurfacing in TKA. Outcomes of knee relevant indicators were analysed. To see the short- and long-term effects, we calculated the data in total and divided the patients who were followed up for ≤ 3 years and ≥ 5 years into two subgroups as well.

**Results:**

Thirty-two trials assessing 6887 knees were eligible. There was a significant difference in terms of reoperation (in total and ≥ 5 years), Knee Society Score (KSS), function score (in total and ≥ 5 years) and noise. While no significant difference was found in the following items: reoperation (≤ 3 years), anterior knee pain (AKP), function score (≤ 3 years), range of motion (ROM), Oxford score, the Knee Injury and Osteoarthritis Outcome Score (KOOS), visual analogue score (VAS), Feller score, patellar tilt and the patients’ satisfaction.

**Conclusions:**

We found that patellar resurfacing could reduce the occurrence of reoperation and noise after surgery, as well as increase the KSS and function score, while it might not influence the outcomes such as AKP, ROM, Oxford score, KOOS, VAS, Feller score, patellar tilt and the patients’ satisfaction. The results are different from our previous finding in the meta-analysis. In conclusion, we prefer patellar resurfacing in TKA.

## Introduction

Total knee arthroplasty (TKA) is one of the most common treatments for patients suffered knee arthritis. Nevertheless, the management of patella during TKA operation still remains controversial [1]. In previous literature reports, there are 3 strategies adopted by different surgeons: patellar resurfacing, patellar nonresurfacing and selective resurfacing [2, 3]. But no consensus on the best management has been reached [4]. The outcome indicators such as Knee Society Score (KSS), function score of KSS, range of motion (ROM), anterior knee pain (AKP) postoperative and the ratio of reoperation are different in various studies [1–5]. The different outcomes of previous studies provide the basis for different choices of patellar resurfacing or not. In 2013, the author conducted a meta-analysis of randomized controlled trials [5]. We found that patellar resurfacing could reduce the risk of reoperation. And in a long-term follow-up, patellar resurfacing might make a difference of KSS. While in other aspects, the benefit of patellar resurfacing was limited. The limitation of our previous meta-analysis is the amount of high-quality randomized controlled trials. Since 2013, more and more RCTs, retrospective studies, even meta-analysis and systematic reviews have been carried out. Still, no clear conclusion has been drawn. In order to see if the result of our previous study has changed and update the latest data, we conducted this update meta-analysis of available RCTs to evaluate the efficacy of patellar resurfacing in TKA. Especially, we evaluated the items as follows: reoperation, AKP, KSS, function score, ROM, Oxford score, the Knee injury and Osteoarthritis Outcome Score (KOOS), visual analogue score (VAS) of pain, Feller score, patella tilt, noise after operation and patients’ satisfaction. Data in total, follow-up no more than 3 years and no less than 5 years were calculated separately.

## Materials and methods

### Identification and eligibility of relevant RCTs

We carried out a literature search using MEDLINE, Ovid and Cochrane Library databases to identify all papers published from January 2013 to May 2020 that evaluated the outcome of patients undertaking TKA with patellar resurfacing or not. Utilizing the methodology of our previously published meta-analysis, the inclusion criteria for this study included [5] (1) English publications, (2) adults undergoing primary TKA, (3) all available RCTs comparing TKAs with and without patellar resurfacing and (4) data for the ratio of reoperation, AKP, knee scores (KSS, function score, Oxford score, KOOS, VAS, Feller score), ROM, patella tilt and noise after operation such as crepitus and patients’ satisfaction. The exclusion criteria were non-English language articles, proceedings of meetings, unpublished data, non-randomized controlled studies, studies of body specimens and researches of TKA but not about patellar resurfacing. To avoid double-counting, multiple publications of the same patient population were pooled as one study. In order to avoid the loss of included literatures, we did not use advanced search strategy this time. And our search words were “patellar resurfacing”, “patellar replacement”, “total knee arthroplasty” and “total knee replacement”. The references of present meta-analysis, systematic reviews and review articles were also been searched from the databases for any missed studies. In the end, we added the 14 RCTs before 2013 in our previous study into this study.

### Outcomes

The researchers sorted the data successively for further analysis as follows: the number of reoperation, the number of patients suffered AKP postoperative, KSS, function score, ROM, Oxford score, KOOS, VAS, Feller score, patella tilt, noise after operation and patients’ satisfaction. To see the short- and long-term effects, we calculated the data in total, follow-up no more than 3 years and no less than 5 years separately.

### Data extraction

Two of the authors extracted the relevant data from each article independently. And a third researcher checked the data against the original information to avoid anthropic mistakes. The extracted data included publication and patients’ characteristics, length of follow-up and numbers of lost to follow-up, knee prosthesis used in TKA and clinical outcomes (AKP, ROM, KSS, Function score and so on).

### Assessment of methodological quality

Included studies were independently rated for methodological quality by two of the authors. Any controversy was cross-checked and resolved by a third author to reach a final consensus. The risks of bias in included studies were accessed using the Cochrane Risk of Bias Tool (Review Manager 5.4). The items assessed were (1) random sequence generation (selection bias), (2) allocation concealment (selection bias), (3) blinding of participants and personnel (performance bias), (4) blinding of outcome assessment (detection bias), (5) incomplete outcome data (attrition bias), (6) selective reporting (reporting bias) and (7) other biases.

### Statistical analysis

The software Review Manager 5.4 (Cochrane Library) was used to analyse the included data. Fixed model or random-effects model were chosen based on the heterogeneity of the studies. A *P* value of < 0.1 and *I*^2^ < 25% were considered suggestive of statistical heterogeneity. The mean difference and 95% confidence (95%CI) were used for continuous data (such as ROM, scores, patellar tilt), while the risk ratios and 95%CI were used for dichotomous outcomes such as reoperation and AKP.

## Results

**Fig. 1:**
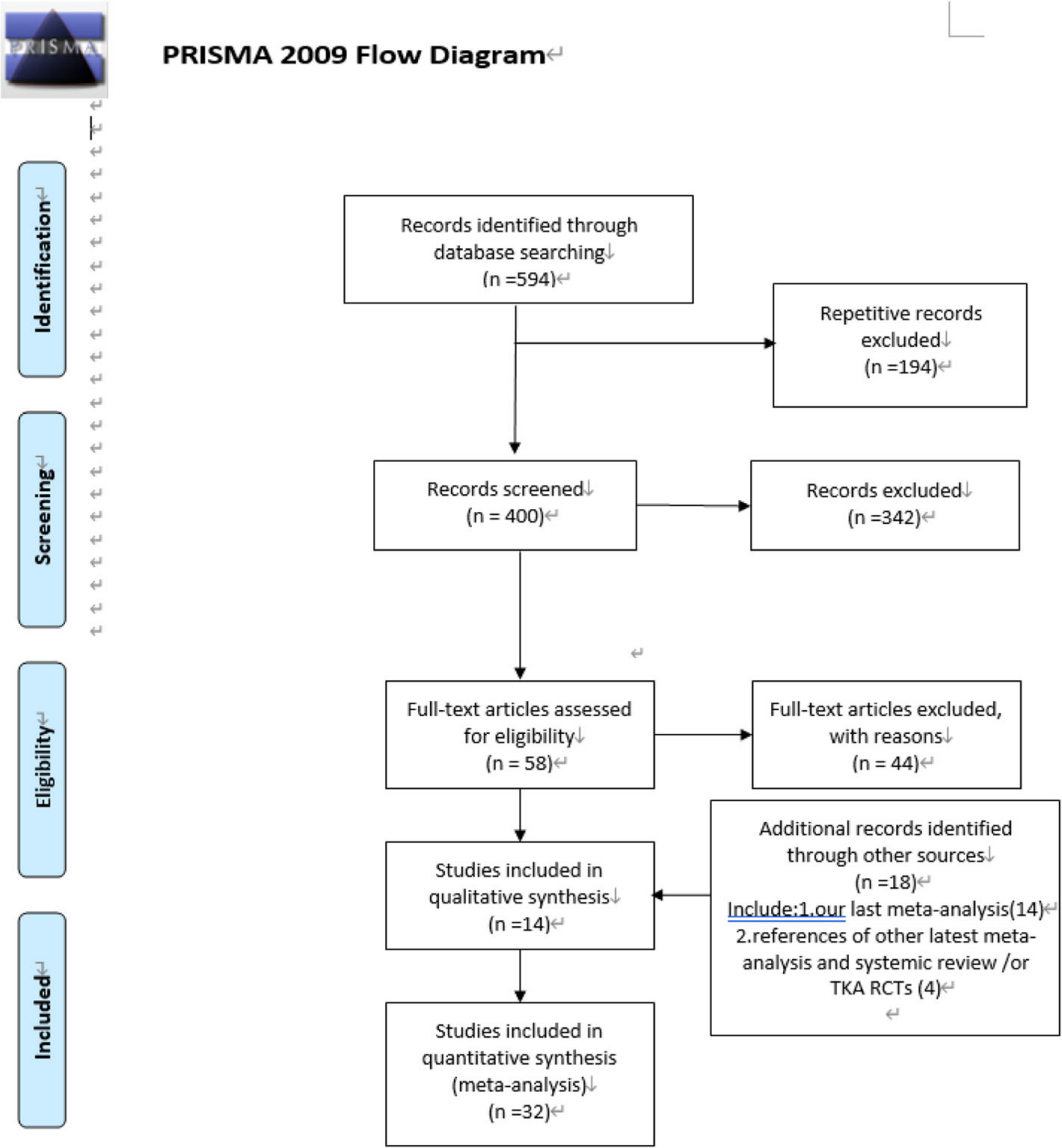
Flow chart of the studies recruited in this meta-analysis

**Fig. 2:**
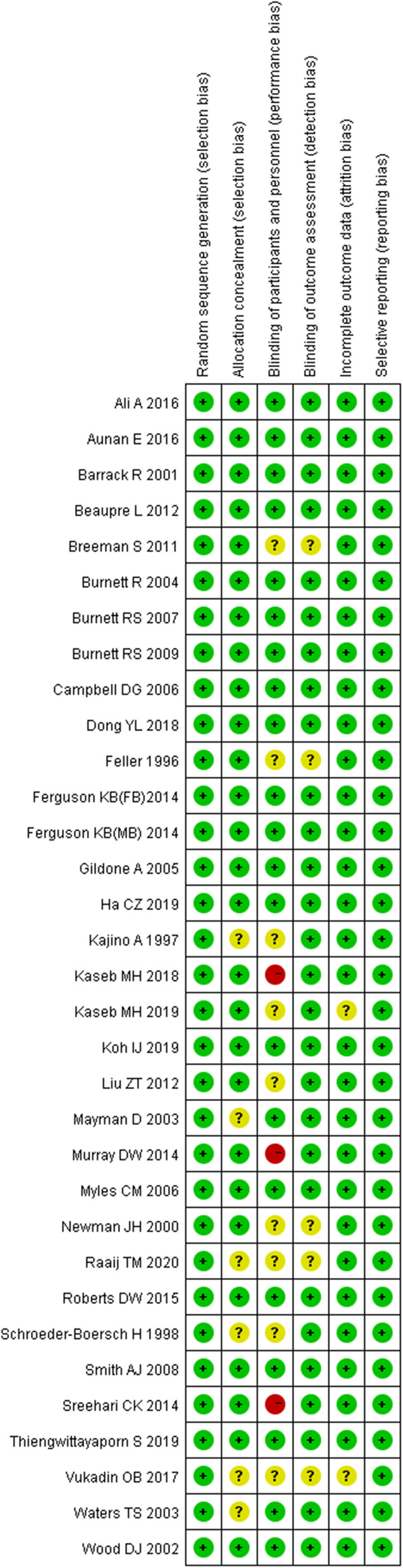
Risk of bias

A flow chart of the recruited studies was shown in Fig. 1. Thirty-two trials [4, 6–36] assessing 6887 knees were selected for inclusion in this meta-analysis. Details on all the studies are shown in Table 1. The methodological quality is shown in Fig. 2 to see the bias risk of each study.

**Table 1:** Characteristics of studies

First author	Journal	Year of publication	Number of knees (RS/NRS)		Mean age (years) (RS/NRS)	Lost to follow-up (knees)	Mean years of follow-up	Outcomes	Level of evidence	Type of knee prosthesis
Feller [6]	JBJS	1996	19	19	70.8	2	2	HSS, patellar score, pain, reoperation	I	PCA (HOWMEDICA)
Kajino [7]	JBJS	1997	26	26	56.1	0	6.6	HSS, pain, muscle strength, patellar tilt	I	Yoshino-Shoji (Biomet)
Schroeder-Boersch [8]	Arch Orthop Trauma Surg	1998	20	20	73/72.2	0	2	KSS, AKP, radiographs, reoperation	I	Duracon (HOWMEDICA)
Newman [9]	Knee	2000	42	42	72/71.2	0	5	AKP, reoperation	I	Kinematic (HOWMEDICA)
Barrack [10]	JBJS	2001	47	46	66.2	11	5–7	KSS, AKP, ROM, satisfaction, reoperation	I	Miller-Galante II (ZIMMER)
Wood [11]	JBJS	2002	92	128	73.7/73.7	22	4	KSS, AKP, reoperation, satisfaction, patellar tilt	I	Miller-Galante II (ZIMMER)
Mayman [12]	JOA	2003	50	50	72/68	29	10	KSS, AKP, reoperation, satisfaction	I	AMK (Depuy)
Waters [13]	JBJS	2003	243	231	69.1	0	5.3	KSS, AKP, satisfaction, ROM, radiographs	I	Press-fit condylar prosthesis (Johnson & Johnson)
Burnett [4]	CORR	2004	42	48	71/69	0	10	KSS, AKP, ROM, satisfaction	I	AMK (Depuy)
Gildone [14]	Acta Orthop Belg	2005	28	28	73.6/74.6	0	2	KSS, daily activities, ROM, satisfaction, AKP	I	NexGen (ZIMMER)
Campbell [15]	JBJS	2006	46	54	71/73	42	10	WOMAC, AKP, radiographs, KSS	I	Miller-Galante II (ZIMMER)
Myles [16]	Clinical Biomechanics	2006	25	25	70	8	2	KSS, WOMAC, VAS, reoperation	I	LCS rotating platform knee replacement (DePuy)
Burnett [17]	CORR	2007	28	28	78	8	10	AKP, ROM, reoperation	I	Miller-Galante II (ZIMMER)
Smith [18]	JBJS	2008	73	86	71.9/71.2	7	4	KSS, AKP, satisfaction, radiographs, reoperation	I	Profix total knee system (Smith & Nephew)
Burnett [19]	JBJS	2009	58	60	65.3/67.1	40	10	ROM, KSS, AKP, satisfaction	II	Miller-Galante II (ZIMMER)
Breeman [20]	JBJS	2011	861	854	70/70	405	5	Oxford score, SF-12, EuroQol-5d, cost-effective, reoperation	I	No mention
Beaupre [21]	BMC Research Notes	2012	21	17	64.9/62	15	10	WOMAC, reoperation, ROM, Rand 36 score	I	Profic total knee system
Liu [22]	Knee	2012	68	64	67.5/68	12	7	KSS, AKP, ROM, radiographs, reoperation	I	Press-fit condylar prosthesis (DePuy)
Ferguson (FB) Feguson (MB) [23]	Knee	2014	88 89	88 87	69.8 70.2	13 13	2	Oxford score, ROM, SF-12 score, reoperation	I	Press-fit condylar prosthesis (DePuy)
Murray [24]	Health Technology Assessment	2014	816	798	70/70	824	10	Oxford score, EuroQol-5d, SF-12 score, reoperation	I	No mention
Sreehari [25]	AO Orthopaedics	2014	75	60	68.1/65.8	0	5	KSS, AKP, ROM, reoperation	I	No mention
Roberts [26]	JOA	2015	178	172	70.2/71.3	236	10.4	KSS, ROM, reoperation, AKP, satisfaction	I	DePuy Sigma
Ali [27]	Acta Orthopaedica	2016	33	36	68/69	5	6	VAS, KOSS, satisfaction, reoperation	I	Triathlon CR
Aunan [28]	Acta Orthopaedica	2016	63	66	70/69	1	3	KOSS, KSS, Oxford score, VAS, satisfaction, reoperation	I	NexGen (ZIMMER)
Vukadin [29]	Acta Chirurgiae Orthopaedicae	2017	30	29	68.1/66	1	2	KSS, Oxford score, reoperation	I	Unknown
Dong [30]	JOA	2018	48	48	67.7	6	3	KSS, Feller score, AKP, reoperation	I	Posterior cruciate stabilizing total knee prostheses (Smith & Nephew)
Kaseb [31]	ABJS	2018	24	26	64.8	0	0.5	KSS, AKP, WOMAC, SF-36 score, ROM, VAS, reoperation	I	Profix (ZIMMER)
Ha [32]	International Orthopaedics	2019	60	60	65.2	12	5	KSS, AKP, satisfaction, reoperation	I	Scorpoo NRG knee prosthesis (Stryker)
Kaseb [33]	ABJS	2019	29	44	68.1/65.75	0	8.68 months	KSS, KOSS	I	NexGen fixed bearing knee prosthesis (ZIMMER)
Koh [34]	KSSTA	2019	49	49	70	0	5	AKP, forgotten score, ROM, WOMAC, reoperation, Feller score, radiograph	I	Posterior stabilized knee system (Lospa)
Thiengwittayaporn [35]	JOA	2019	41	39	68.2/68.2	4	1	KSS, AKP, Oxford score, ROM, patellar score, patellar tilt	I	Legion PS (Smith & Nephew)
Raaij [36]	J Knee Surg	2020	21	21	Unknown	0	2	HSS, KSS, KOSS, reoperation	I	Unknown

*RS* Resurfacing, *NRS* Nonresurfacing, *AKP* Anterior knee pain, *KSS* Knee Society Score, *HSS* Hospital for special surgery, *ROM* Range of motion, *WOMAC* Western Ontario McMaster Osteoarthritis Index, *KOOS* Knee Injury and Osteoarthritis Outcome Score

### Reoperation

**Fig. 3:**
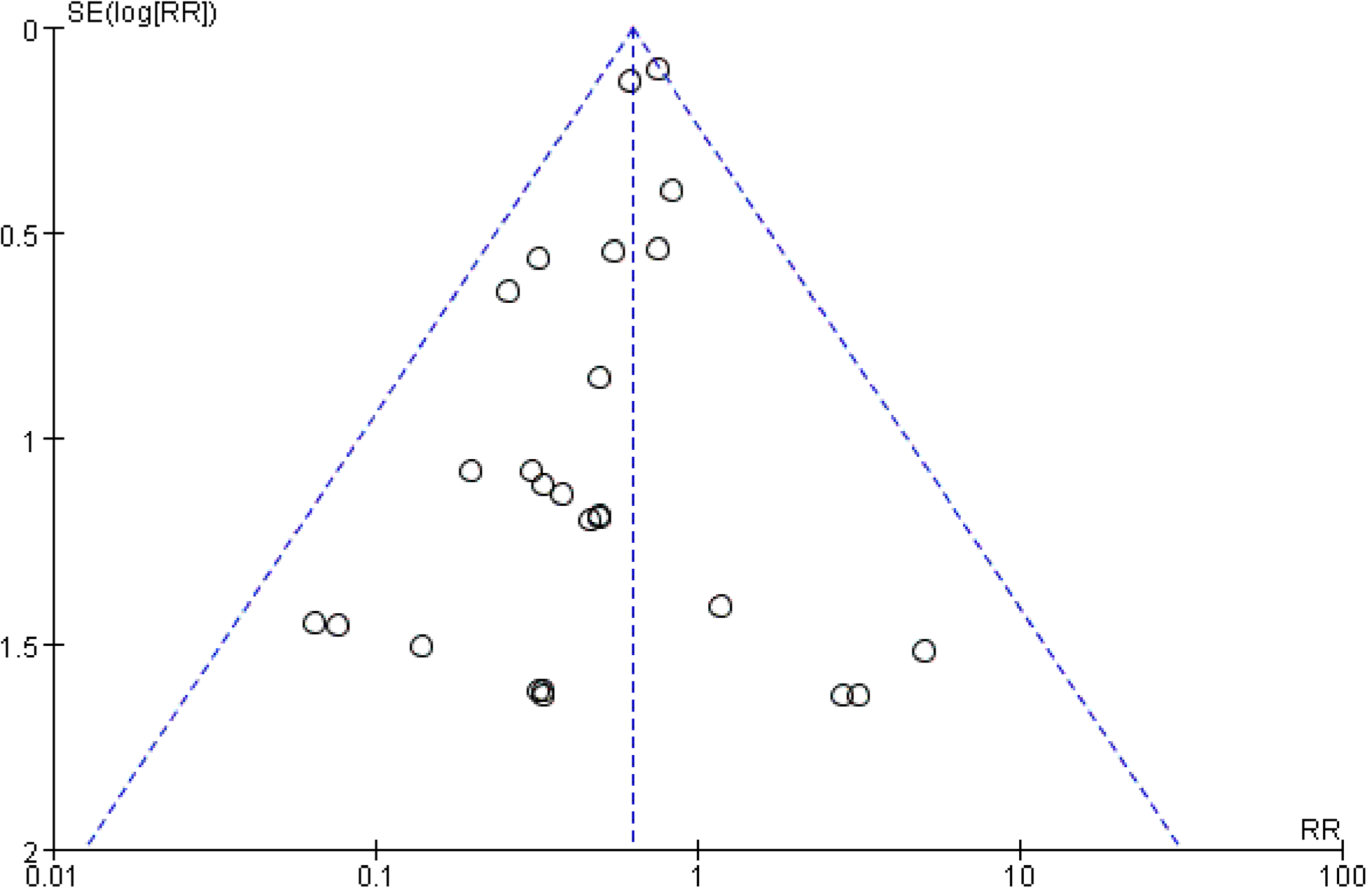
Funnel plot

**Fig. 4:**
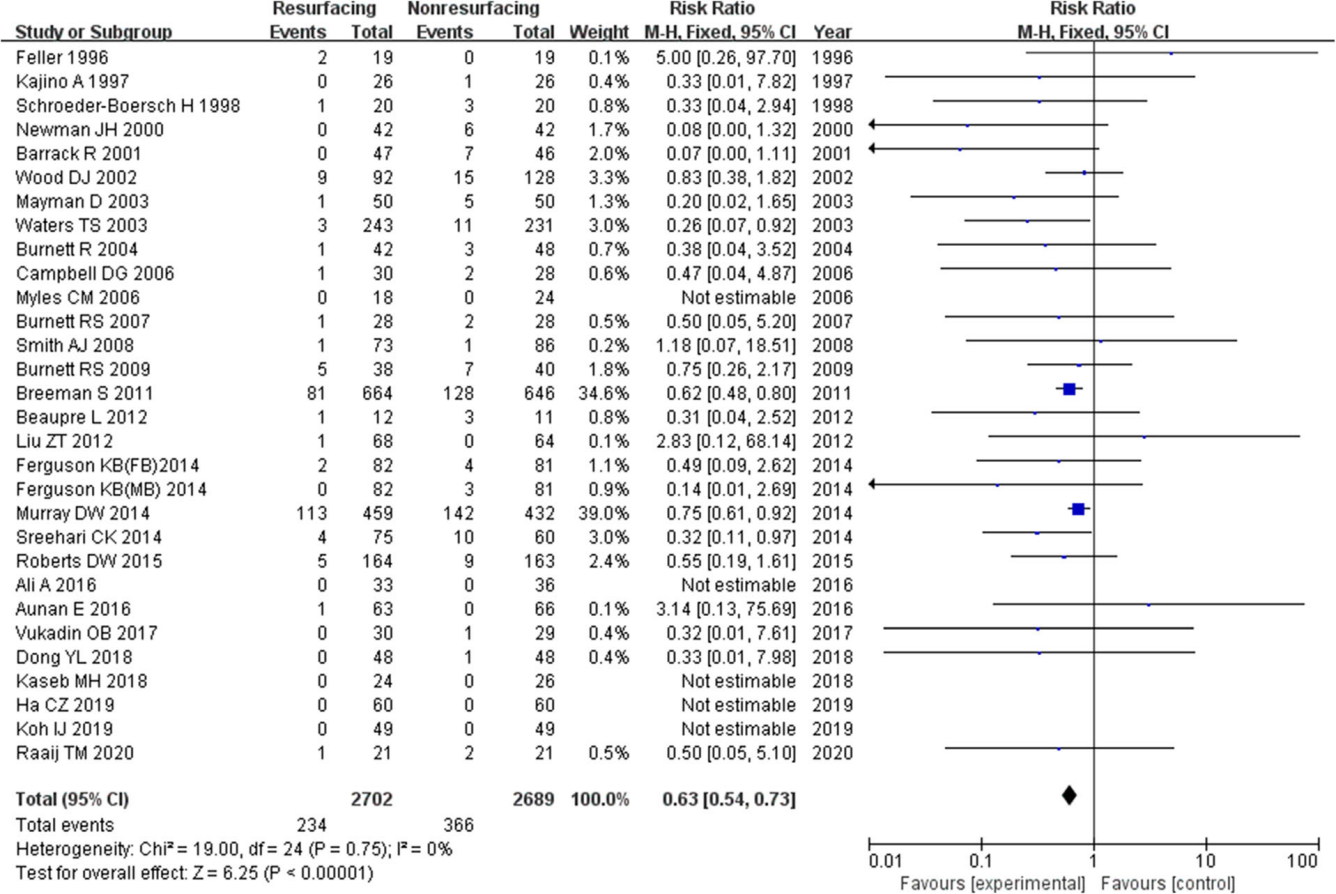
Reoperation in total

**Fig. 5:**
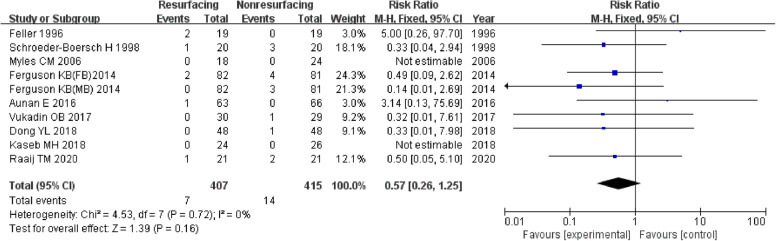
Reoperation (≤ 3 years)

**Fig. 6:**
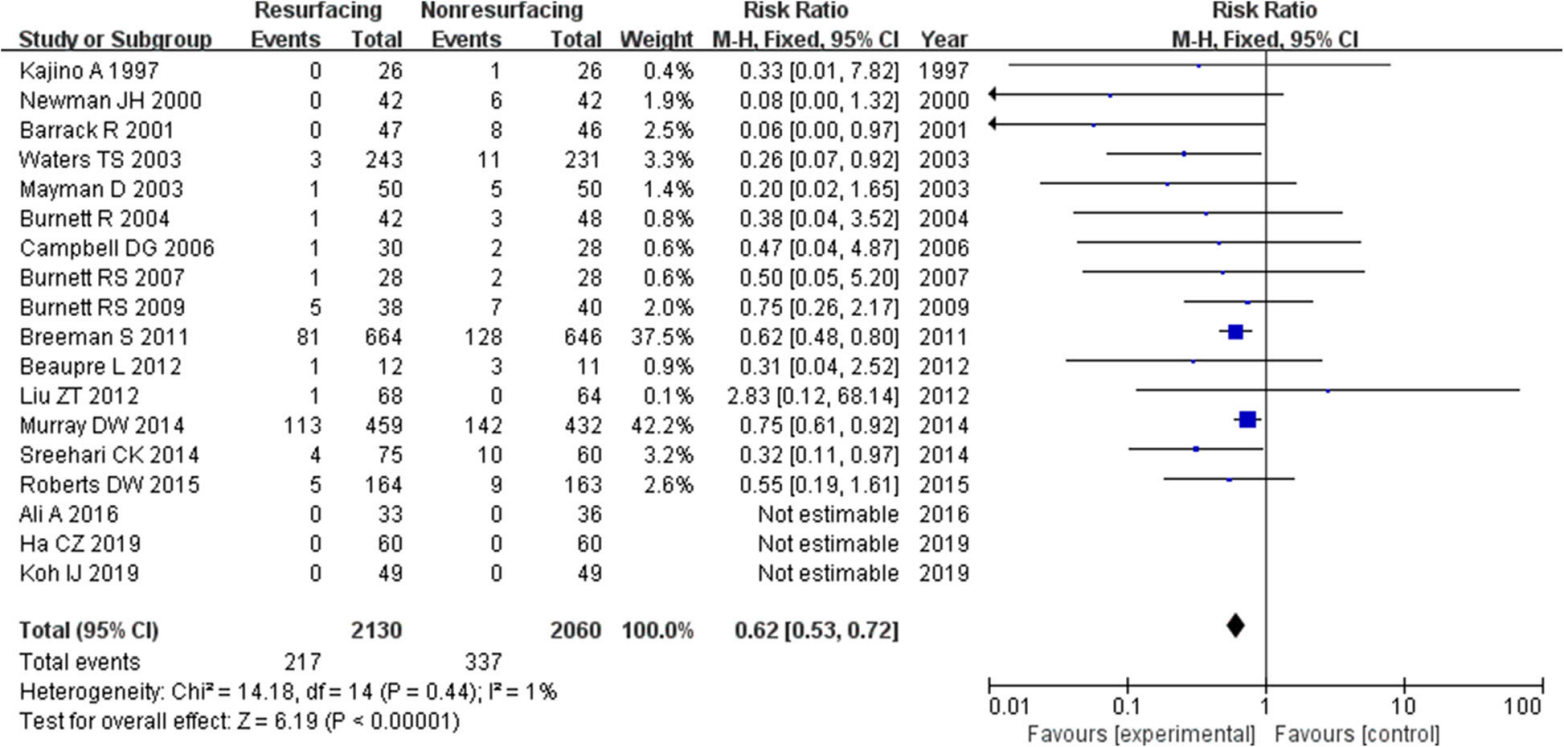
Reoperation (≥ 5 years)

A total of 5391 knees were included when comparing the ratio of reoperation for any reason postoperative which was described in 30 studies. The funnel plot showed low publication bias of the 30 researches (Fig. 3). The risk ratio (RR) (RR = 0.63, 95%CI 0.54~0.73, *P* < 0.00001, Fig. 4) implied that there was a significant difference between the two groups. And the test for homogeneity was not significant (*P* = 0.75). We could draw a conclusion that patellar resurfacing could reduce the reoperation rate postoperative. In addition, we analysed reoperation of 10 studies with a follow-up of no more than 3 years. The test for homogeneity showed no significant difference (*P* = 0.72). No significant difference was found between the reoperation data of the 2 groups (RR = 0.57, 95%CI 0.26~1.25, *P* = 0.16, Fig. 5). Eighteen studies included data no less than 5 years. The test for homogeneity showed no significant difference (*P* = 0.44). A significant difference was found between the reoperation data of the 2 groups (RR = 0.62, 95%CI 0.53~0.72, *P* < 0.00001, Fig. 6).

### Anterior knee pain

**Fig. 7:**
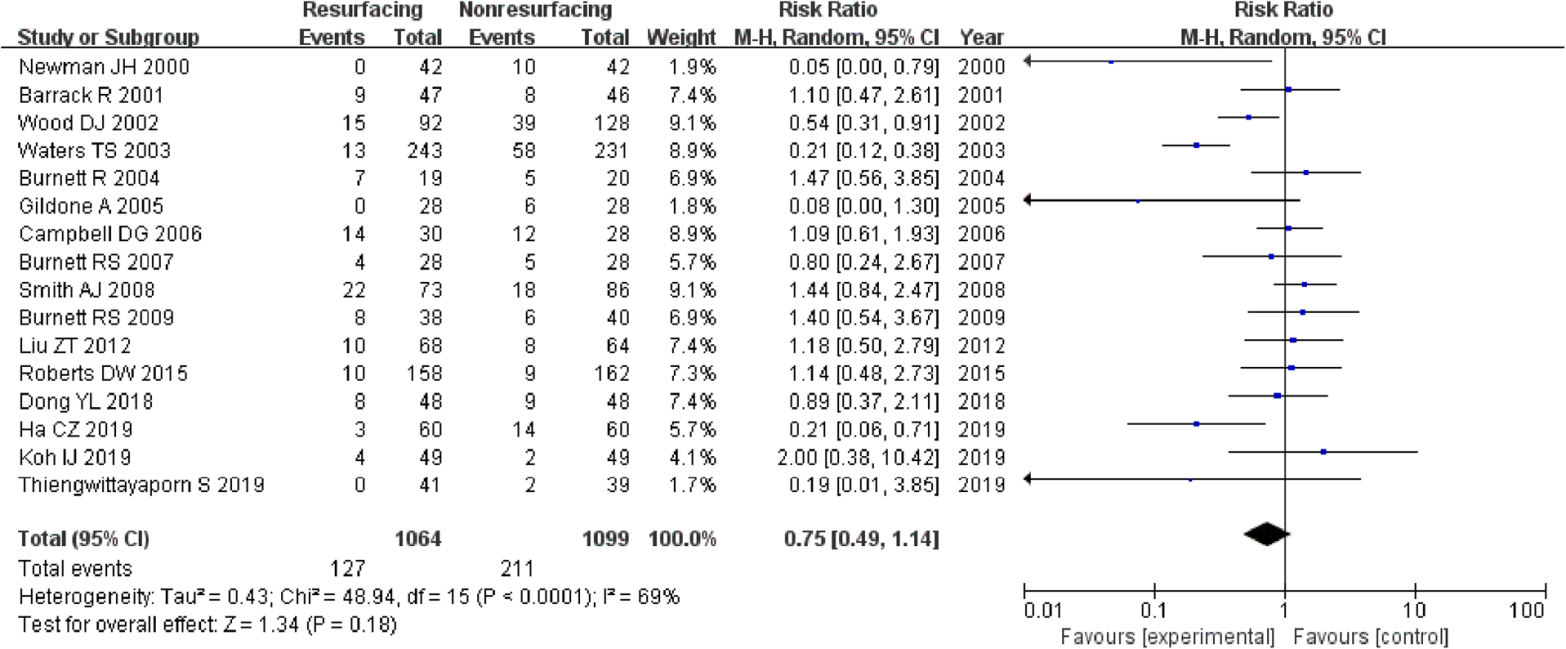
AKP in total

**Fig. 8:**
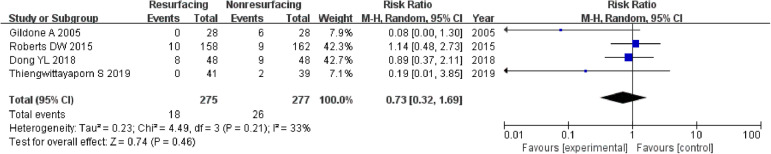
AKP (≤ 3 years)

**Fig. 9:**
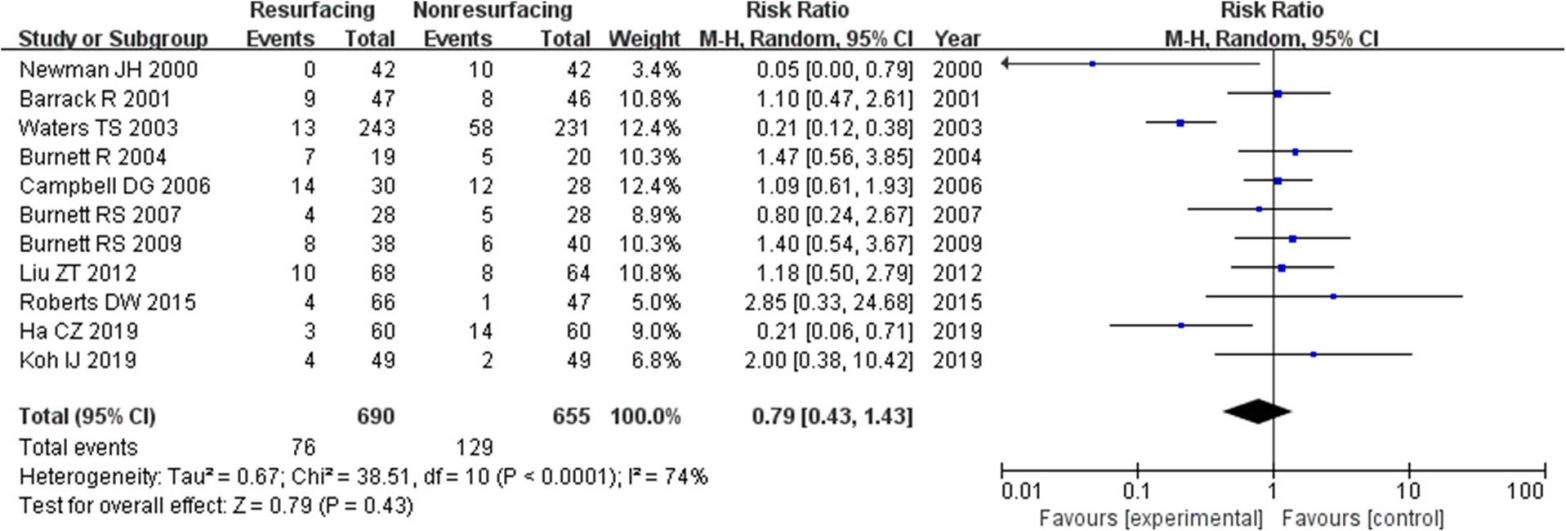
AKP (≥ 5 years)

Sixteen studies included 2163 knees reported on the incidence of AKP. The risk ratio for AKP was not significant in total (RR = 0.75, 95%CI 0.49~1.14, *P* = 0.18, Fig. 7), while the test for homogeneity showed significant difference (*P* < 0.00001). Four studies reported on data ≤ 3 years (RR = 0.73, 95%CI 0.32~1.69, *P* = 0.46, Fig. 8), and 11 studies reported on data ≥ 5 years (RR = 0.79, 95%CI 0.43~1.43, *P* = 0.43, Fig. 9). All the analyses showed that there was no remarkable difference in AKP between the resurfacing group and the nonresurfacing group.

### Knee Society Score

**Fig. 10:**
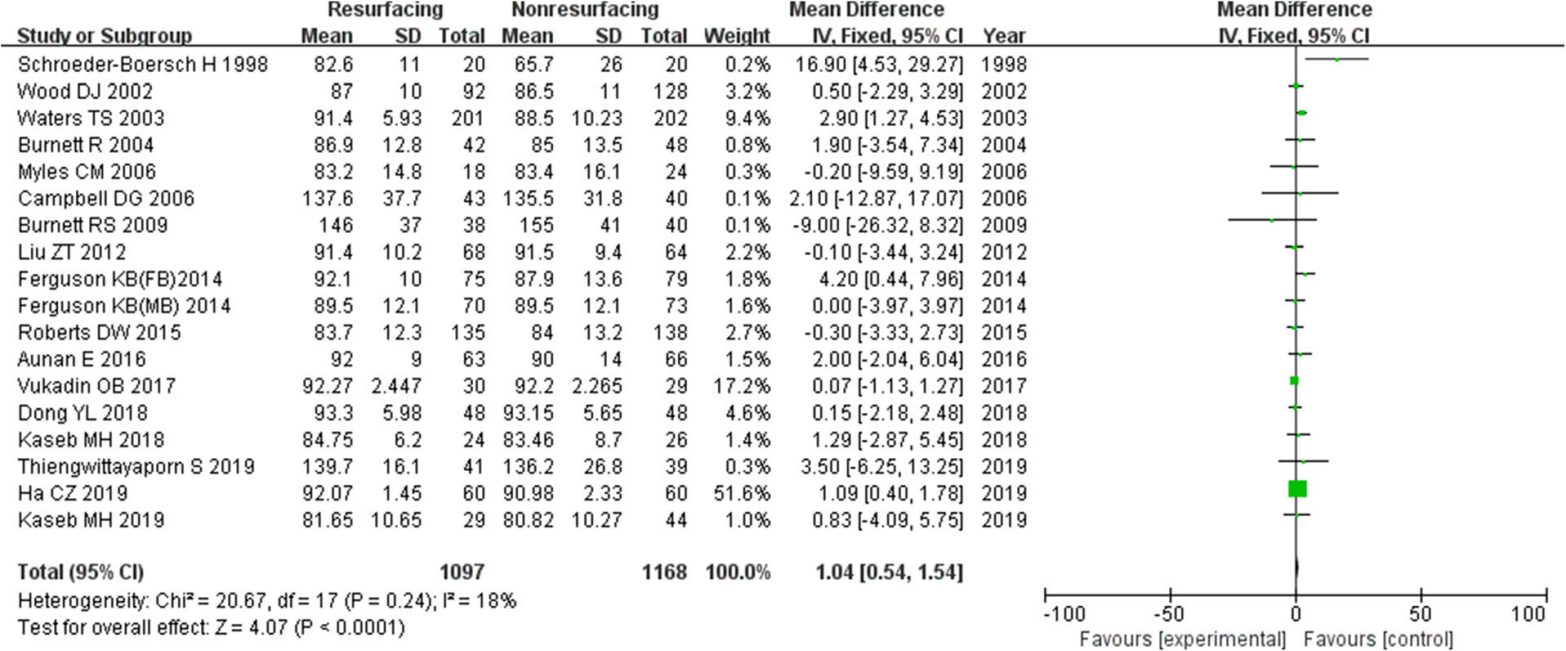
KSS in total

**Fig. 11:**
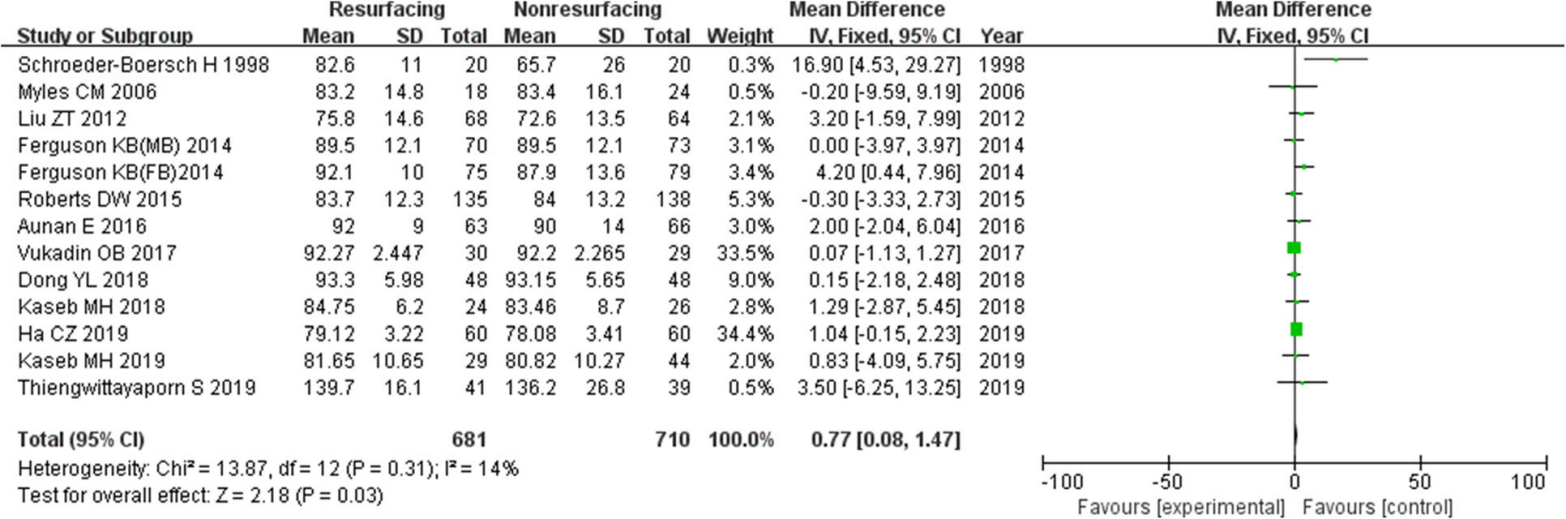
KSS (≤ 3 years)

**Fig. 12:**
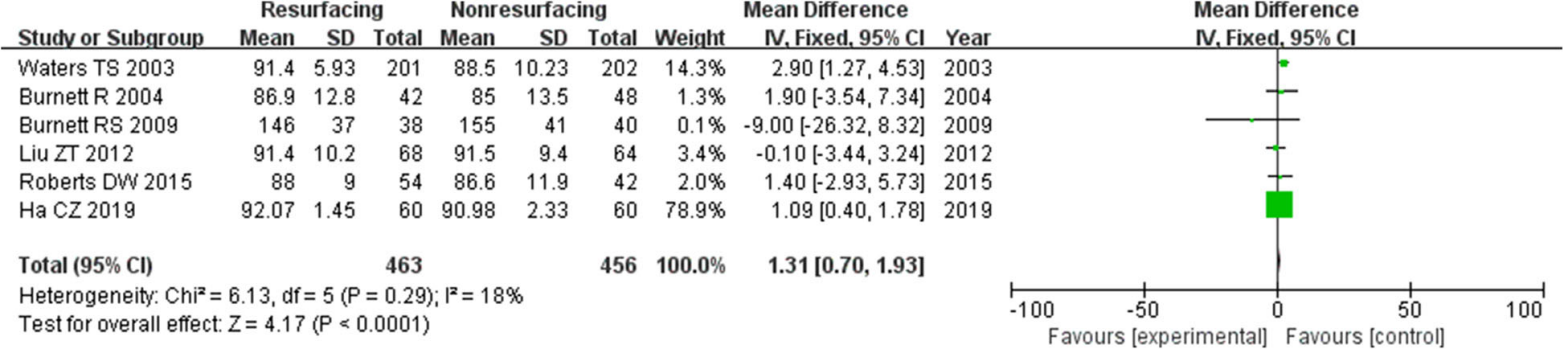
KSS (≥ 5 years)

Eighteen studies included 2265 knees reported on the necessary data of KSS. The mean difference (MD) for KSS was significant in total (MD = 1.04, 95%CI 0.54~1.54, *P* < 0.00001, Fig. 10). And the test for homogeneity showed no significant difference (*P* = 0.24). Thirteen studies reported on data ≤ 3 years (MD = 0.77, 95%CI 0.08~1.47, *P* = 0.03, Fig. 11), and 6 studies reported on data ≥ 5 years (MD = 1.31, 95%CI 0.70~1.93, *P* < 0.00001, Fig. 12). All the analyses showed that there was a significant difference of KSS between the resurfacing group and the nonresurfacing group. It seemed that the patellar resurfacing group might get higher KSS scores after primary surgery.

### Function score

**Fig. 13:**
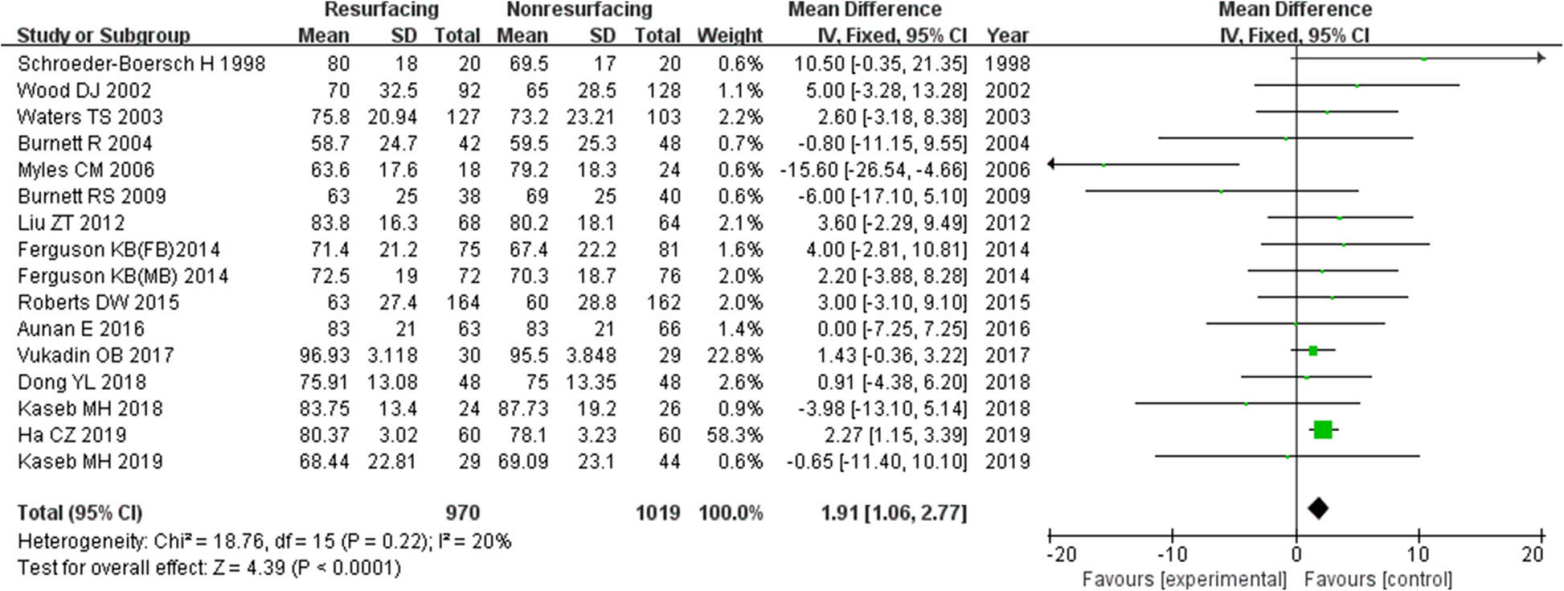
Function score in total

**Fig. 14:**
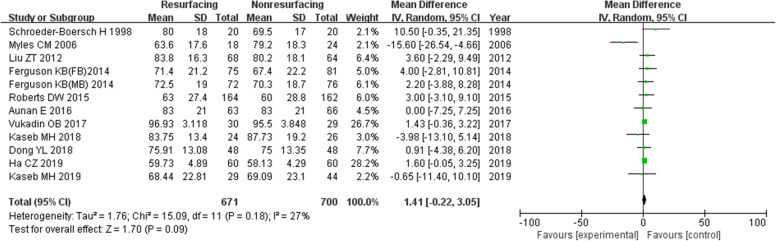
Function score (≤ 3 years)

**Fig. 15:**
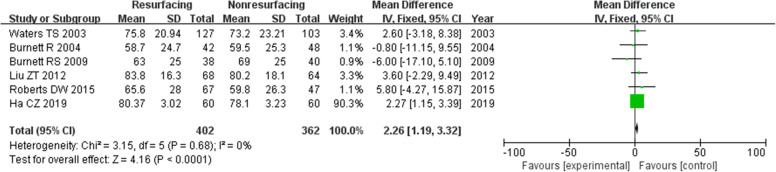
Function score (≥ 5 years)

The function score was the function component of KSS. In our study, 16 studies of 1989 knees showed a function score. There was a significant difference between the two groups in total and for ≥ 5 years, while the difference ≤ 3 years was not significant (in total MD = 1.91, 95%CI 1.06~2.77, *P* < 0.00001, Fig. 13; ≤ 3 years MD = 1.41, 95%CI − 0.22~3.05, *P* = 0.09, Fig. 14; ≥ 5 years MD = 2.26, 95%CI 1.19~3.32, *P* < 0.00001, Fig. 15). The *P* value of heterogeneity was 0.22 showing low heterogeneity.

### Range of motion

**Fig. 16:**
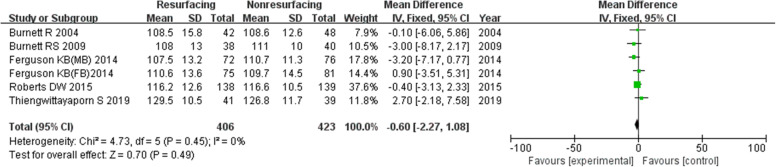
ROM in total

Six studies included 829 knees reported on necessary data of ROM. It seemed that there was no significant difference between the two groups after operation (in total MD = − 0.60, 95%CI − 2.27~1.08, *P* = 0.49, Fig. 16; ≤ 3 years MD = − 0.33, 95%CI − 2.19~1.52, *P* = 0.72; ≥ 5 years MD = − 0.34, 95%CI − 3.21~2.52, *P* < 0.81). The *P* value of heterogeneity was 0.45 showing low heterogeneity.

### Oxford score

**Fig. 17:**
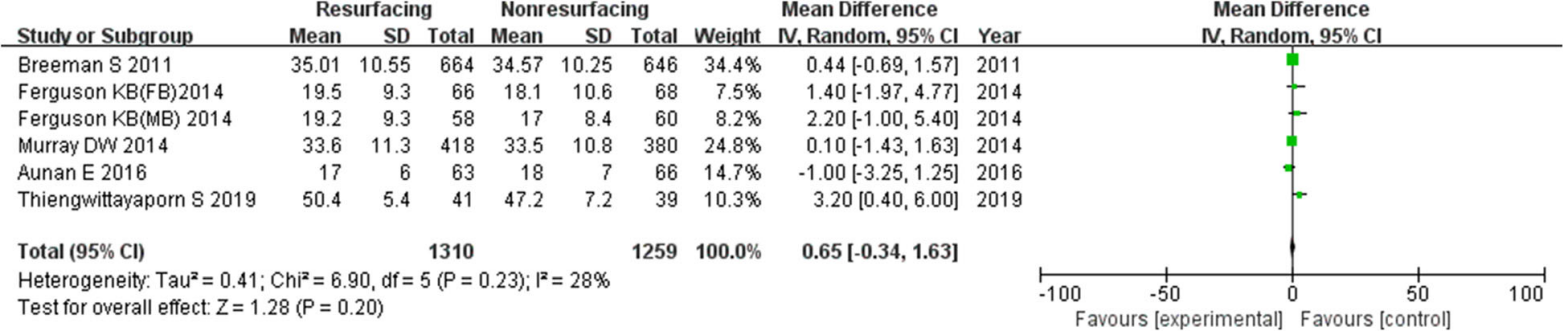
Oxford score in total

Six studies included 2569 knees reported on necessary data of Oxford score. It seemed that there was no significant difference between the resurfacing group and the nonresurfacing group (in total MD = − 0.65, 95%CI − 0.34~1.63, *P* = 0.20, Fig. 17; ≤ 3 years MD = 0.51, 95%CI − 0.40~1.41, *P* = 0.27; ≥ 5 years only 2 studies).

### The Knee Injury and Osteoarthritis Outcome Score

**Fig. 18:**
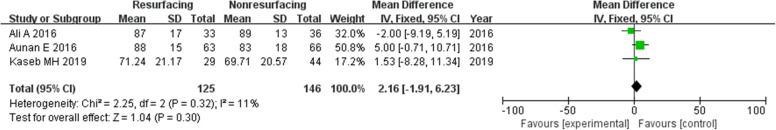
KOOS in total

Three studies included 277 knees reported on necessary data of KOOS score. There was no significant difference between the two groups (in total MD = 2.16, 95%CI − 1.91~6.23, *P* = 0.30, Fig. 18).

### VAS

Three studies included 217 knees reported on necessary data of VAS score. There was no significant difference between the two groups (in total MD = − 0.10, 95%CI − 0.42~0.22, *P* = 0.55).

### Feller score

Three studies included 274 knees reported on necessary data of Feller score. There was no significant difference between the two groups (in total MD = 0.31, 95%CI − 0.8~1.43, *P* = 0.59).

### Patellar tilt

Five studies included 444 knees reported on necessary data of patellar tilt angle. There was no significant difference between the two groups (in total MD = − 0.03, 95%CI − 0.36~0.30, *P* = 0.86).

### Noise after operation

Five studies included 503 knees reported on necessary data of the appearance of noise such as clunk, crepitus and so on. There was a significant difference between the two groups (in total RR = 0.47, 95%CI 0.32~0.68, *P* < 0.00001). It seemed that patellar resurfacing might reduce the occurrence of noise postoperative.

### Patients satisfaction

**Fig. 19:**
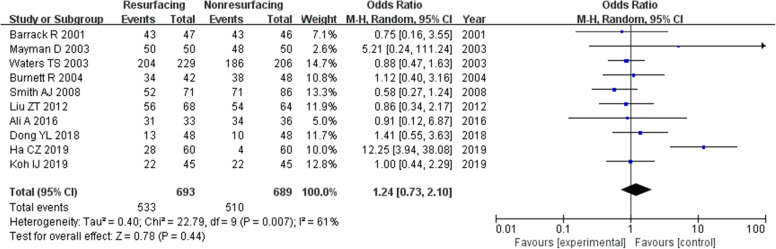
Satisfaction in total

We got 10 studies of 1382 knees showing the number of patients satisfied with the operation (Fig. 19). It seemed that there was no significant difference between the two groups (in total RR = 1.24, 95%CI 0.73~2.10, *P* = 0.44), while the *P* value of heterogeneity was 0.007, which showed high heterogeneity.

## Discussion

In this meta-analysis, 32 randomized controlled trials assessing 6887 knees were adopted. We analysed the data by different statistical indicators and follow-up periods (3 subgroups: in total, ≤ 3 years and ≥ 5 years ). In summary, we found that there was a significant difference between the two groups in terms of reoperation (in total and ≥ 5 years), KSS (all 3 subgroups), function score (in total and ≥ 5 years) and noise postoperation, while no significant difference was found between the resurfacing and the nonresurfacing group in the following items: reoperation (≤ 3 years), AKP (all 3 subgroups), function score (≤ 3 years), ROM, Oxford score, KOOS, VAS, Feller score, patellar tilt and the patients’ satisfaction. We could conclude that patellar resurfacing might reduce the occurrence of reoperation and noise after surgery, as well as increase the KSS and function score. Especially, the noise syndrome, such as clunk, crepitus and so on, was seen in some of the latest studies. Ha et al. and we got the same conclusion that patellar resurfacing might reduce the ratio of noise after primary TKA surgery [32], while the relationship between noise and other scores was not clear. The results of reoperation and function score for ≤ 3 years were not significantly different in our study. This indicated that the follow-up periods might affect the outcome indicators. Nevertheless, our study showed that patellar resurfacing might not influence the results such as AKP, ROM, Oxford score, KOOS, VAS, Feller score, patellar tilt and the patients’ satisfaction compared with nonresurfacing. Especially for the AKP result, the findings of our study are close to that in Teel et al.’s meta-analysis and different from Migliorini et al.’s study [37, 38].

During the implementation process of our study, we searched a great many literatures comparing patellar resurfacing versus nonresurfacing in TKA. Though the non-RCTs were excluded from this study, we still read the literature as well as previous meta-analysis and systemic reviews thoroughly. Twenty-two non-RCTs [39–60] and 15 meta-analysis or systemic reviews [2, 5, 37, 38, 61–71] were singled out ever since 2013. In the non-RCT studies, nine stated that there was no difference between patellar resurfacing and nonresurfacing. Twelve preferred patellar resurfacing for the reasons such as better results in mid-term evaluation, lower rate of reoperation, lower incidence of noise, lower complication rate, higher patients satisfaction, lower AKP rate and higher ROM result, while in Crawford et al.’s study [58], it seemed that TKA with patellar resurfacing had a higher incidence of manipulation under anaesthesia than nonresurfacing. Coory et al. [57] conducted a study of 570,735 TKAs followed up for 17 years from 1999 to 2017. They found that patellar resurfacing might reduce the rate of revision for both MS and PS knees, and the rate of reoperation for MS knees was the lowest. Especially, in Feng et al.’s two studies [41, 59] of Chinese people, there was no significant difference between resurfacing and nonresurfacing. We speculated that the race might influence the outcomes of patellar resurfacing. Furthermore, amongst the 15 meta-analysis or systemic reviews, the studies of Cheng et al. [61], Jonbergen et al. [62] and Grassi et al. [70] indicated that patellar resurfacing had no advantages with nonresurfacing. Petersen et al. [64] believed that the functional causes of AKP might be distinguished from mechanical causes, while Arirachakaran et al. [67] indicated that patellar denervation might improve the knee function but does not improve pain compared with patellar resurfacing. In the researches of Longo et al. [4], Tang et al. [71], Migliorini et al. [38] and Teel et al. [37], patellar resurfacing got the advantage in the following aspects: better KSS and function scores and lower reoperation rate. These 4 studies were carried out in the last 3 years including the latest RCTs preceding 2018. It showed the necessity for high-quality RCT research.

Some limitations of this study should be acknowledged: (1) data of RCTs were not fully reported; (2) the sample size and follow-up time were not close in different RCTs, which might cause heterogeneity; (3) some studies did not provide standard deviation, and this might cause the loose of some studies; (4) still we could not get the full text of few RCTs, which might cause bias; (5) the kind of knee prosthesis and the race of the patients may affect the clinical outcomes as well as the result of this meta-analysis; and (6) the skills of surgeons might also influence the outcomes of each group, so a standard to assess the surgery skills was needed to avoid surgeon bias.

Compared with the above literatures, our study analysed more outcome indicators, included the latest RCTs until May 2020 and got updated conclusions. In addition, we suggested further research directions as follows: (1) more high-quality RCTs of large sample size and long-term follow-up (such as 5–10 years or even longer), (2) more RCTs about the influence of TKA prosthetic type on outcomes of patellar resurfacing, (3) more studies about different patellar resurfacing outcomes of osteoarthritis and rheumatoid arthritis, (4) RCTs about the influence of different grade of patellofemoral arthritis, (5) RCTs focus on the relationship between radiograph measurement (such as angles) and outcome indicators (such as complication, scores and pain), and (6) studies about different race.

## Conclusion

This study is an update meta-analysis of our previous study. We included more high-quality RCTs and analyse more outcomes. Subgroup analysis of different follow-up time is conducted. In conclusion, we find that patellar resurfacing could reduce the occurrence of reoperation and noise after surgery, as well as increase the KSS and function score, while it may not influence the results such as AKP, ROM, Oxford score, KOOS, VAS, Feller score, patellar tilt and the patients’ satisfaction. The result is different from our previous meta-analysis. As a result of this new study, we prefer patellar resurfacing in TKA, while, still, more high-quality RCTs are expected eagerly.

## Data Availability

Yes

## Abbreviations

TKA: Total knee arthroplasty
KSS: Knee society score
AKP: Anterior knee pain
ROM: Range of motion
RCTs: Randomized controlled trials
KOOS: Knee injury and osteoarthritis outcome score
VAS: Visual analogue score
